# Anterior Palatal Fistula Formation after Le Fort I Osteotomy in Conventional Orthognathic Surgery

**DOI:** 10.1155/2023/9038781

**Published:** 2023-08-03

**Authors:** Saeed Nezafati, Tannaz Pourlak

**Affiliations:** Department of Oral and Maxillofacial Surgery, School of Dentistry, Tabriz University of Medical Sciences, Tabriz, Iran

## Abstract

The prevalence rate of maxillary ischemic complications following Le Fort I osteotomy was estimated to be about 1%. Understanding the local and systemic factors affecting maxillary perfusion before, during, and after the surgery can prevent the development of these complications. The present study investigated a case of anterior palatal fistula following the classic Le Fort I osteotomy.

## 1. Introduction

Maxillary orthognathic surgery is a predictable, safe, and stable procedure [1]. Despite all the advances made in orthognathic surgery, various complications have been reported following this surgery, including maxillary sinusitis, the loss of dental vitality, sensory problems, aseptic necrosis, and vascular problems, such as hemorrhage or arteriovenous fistulas [2, 3]. The prevalence rate of these complications is approximately between 6% and 9% [4, 5]. The prevalence rate of ischemic complications following Le Fort I osteotomy was estimated to be about 1% [2]. Although direct maxillary perfusion is provided by descending palatal arteries, even in the case of bilateral ligation of this artery during maxillary down fracture, the maxillary blood supply from the collateral network, including the ascending pharyngeal, ascending palatine arteries in the soft palate, and a mucosal rich network could be provided [6–10].

**Figure 1:**
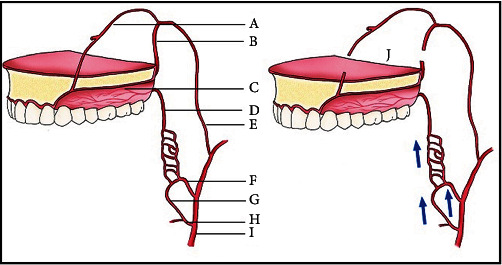
Normal blood flow after Le Fort 1 osteotomy. Blood supply of the maxilla. (A) Nasopalatine artery. (B) Descending palatine artery. (C) Greater palatine artery. (D) Lesser palatine artery. (E) Maxillary artery. (F) Ascending pharyngeal artery. (G) Ascending palatine artery. (H) Facial artery. (I) External carotid artery. (J) Le Fort I down fracture.

Figure 1 shows normal blood flow after Le Fort I osteotomy [11].  Table 1 shows both local and systemic risk factors effective in increasing the risk of improper blood supply of the maxilla after Le Fort I osteotomy [11].

## 2. Case Report

**Figure 2:**
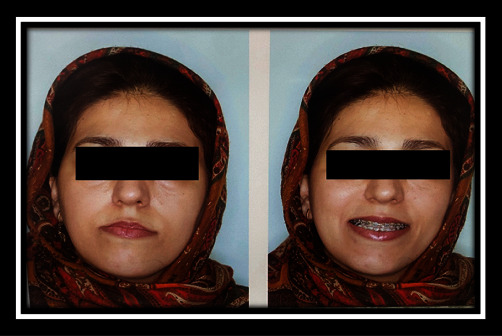
Frontal view before surgery.

**Figure 3:**
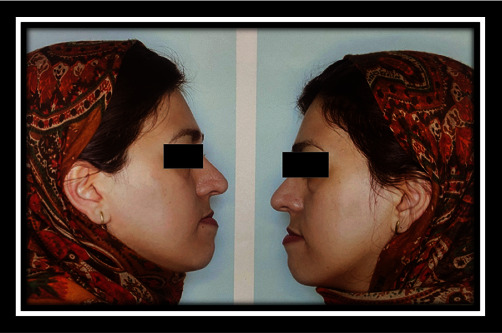
Profile view before surgery.

**Figure 4:**
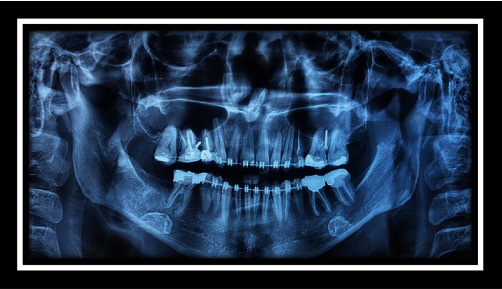
Panoramic view before surgery.

**Figure 5:**
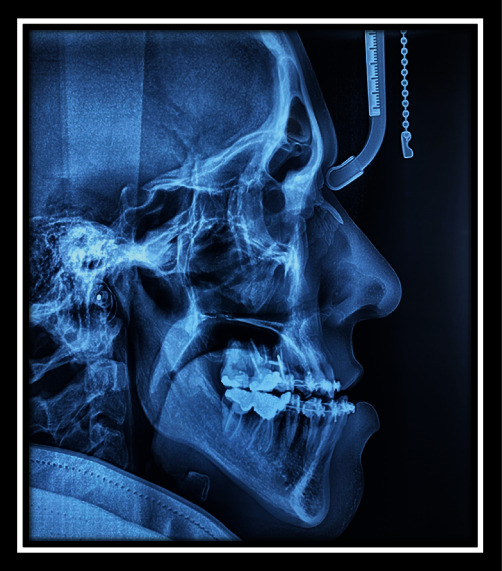
Cephalometric view before surgery.

**Figure 6:**
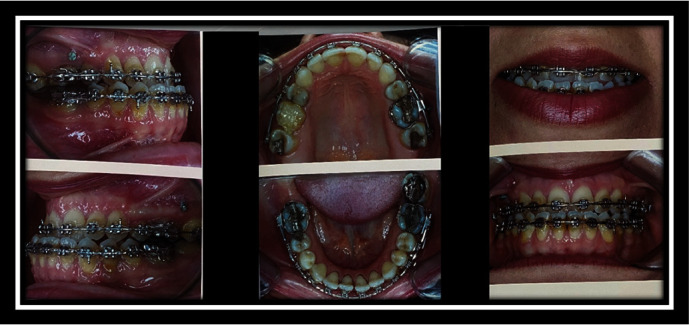
Occlusion before surgery.

**Figure 7:**
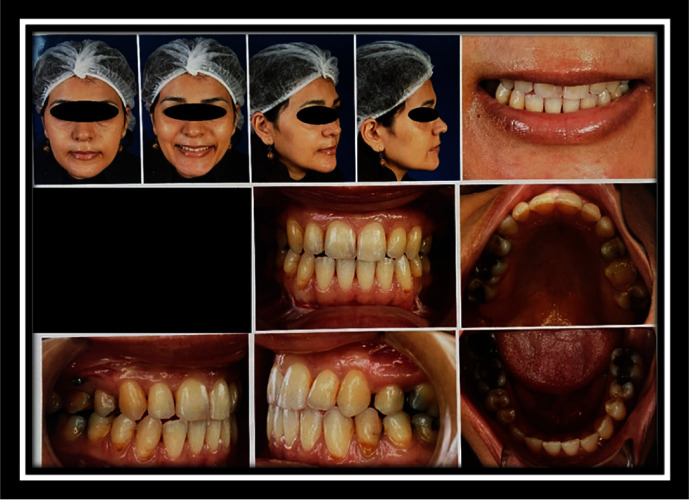
Photography after surgery.

**Figure 8:**
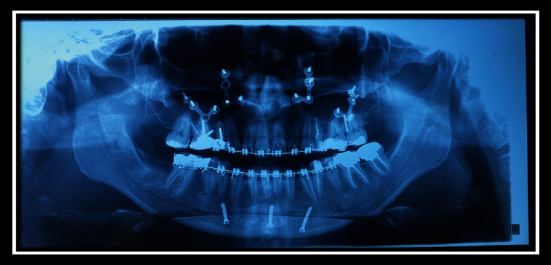
Panoramic view after surgery.

**Figure 9:**
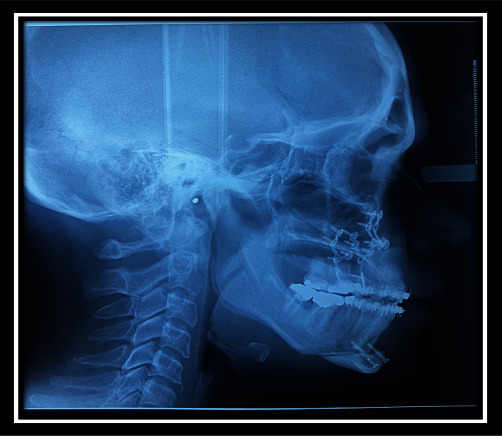
Cephalometric view after surgery.

**Figure 10:**
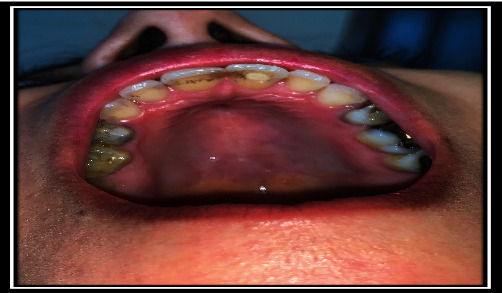
Clinical fistula view.

**Figure 11:**
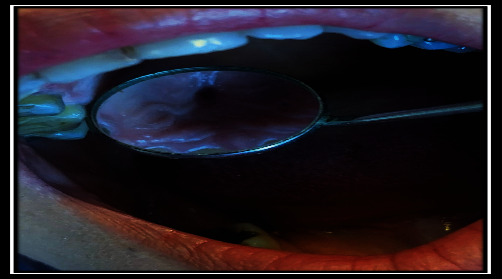
Clinical fistula view.

**Figure 12:**
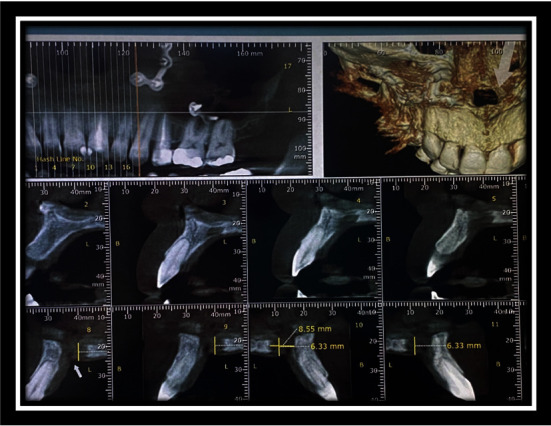
CBCT after fistula formation.

In the present study, the patient was a 35-year-old woman without any systemic problem and with the chief complaint of maxillary retrusion who was referred by the orthodontist for orthognathic surgery. Clinical and cephalometric analyses for this case revealed maxillary and chin retrognathism and chin excessive elongation. The treatment plan considered was Le Fort I osteotomy and 6 mm maxillary advancement along with shortening and advancement genioplasty of the chin as about 3 mm. The classic incision of Le Fort I osteotomy was performed. Le Fort I osteotomy and maxillary advancement were performed with no particular problem. Although cauterization of large arteries was not performed, and minor bleeding in the area of the nasopalatine artery was controlled with electrosurgery cauterization (including device name, power, and duration of usage). The fixation was performed with four mini-plate. Genioplasty was also performed with the preservation of the mental nerve, and fixation was performed with three 15-mm screws. Intraoperative bleeding was low, and recovery was done without causing any problems. After surgery, the result was excellent, and the patient and her companions were very satisfied. By passing 4 weeks from the surgery, the patient used training elastic, and there was no problem during the post-operative period. The patient's oral hygiene was in moderate to good condition. In the fourth week of the visit, the patient complained of swelling of the palate behind the upper first incisors, and she had noticed it from the second week but had forgotten to inform the surgeon. In the clinical examination, a slight fluctuant hemorrhagic swelling on the incisive papilla was observed, and its clinical diameter was approximately 5–10 mm. Notably, no pain or discharge was seen. The patient did not mention trauma or consumption of any kind of solid foods (her statements). Conservative treatment, including rinsing with serum and taking antibiotics was then prescribed. By passing 6 days from the visit, the patient reported the presence of a sharp piece, and a small sharp piece was excluded earlier. At the patient's visit, another piece of bone with a size of 2 mm × 2 mm was removed, and the swelling was drained as well. The recovery was performed within 2 weeks, but a 2–4 mm hole remained at the swelling site, which was not a problem in the first few days, but in the following weeks, regurgitation of fluid from the mouth to the nose was reported by the patient. The patient complained of bad odor and taste along with a slight change in her voice. The treatment continued, and cone beam computer tomography was requested one month later. As well, 5–6 mm defects were seen in the bone. The diagnosis of bone and soft tissue necrosis was made due to cauterization. The corrective surgery for the defect was postponed for 6 months (Figures 2–12).

## 3. Surgical Approach

**Figure 13:**
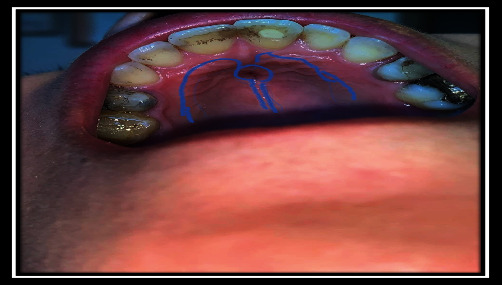
Design for fistula closure.

**Figure 14:**
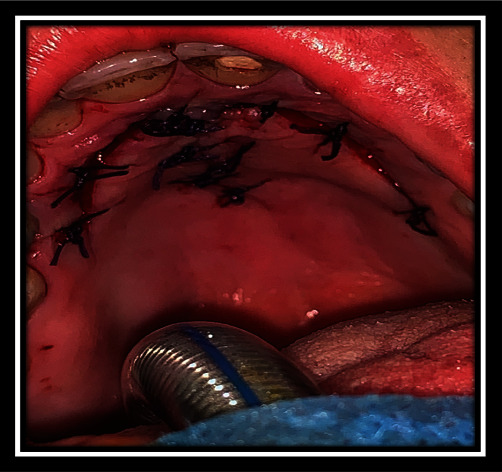
Closure in operative room.

**Figure 15:**
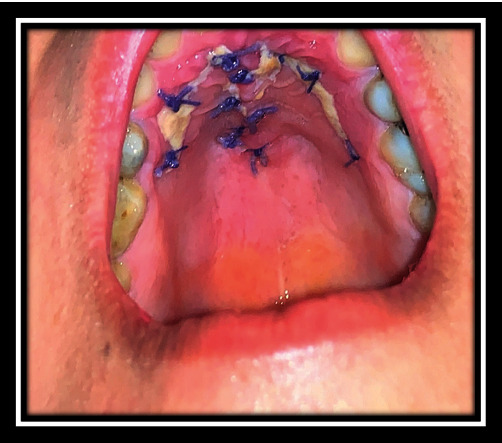
Follow-up after 10 days.

**Figure 16:**
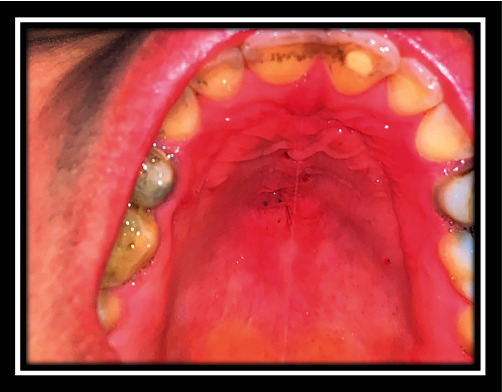
Follow-up after 6 months.

Finally, to determine the extent of possible necrosis, the surgery was performed under general anesthesia using an oral tube and Davis Gag. The von-Langenbeck flap was done, and the closing of the fistula was performed as shown in the figures. Suturing with 4-0 Vicryl was performed as well (Figures 13, 14). The patient's post-operation healing stage and six-month follow-up period were without any problems (Figure 15, 16).

## 4. Discussion

According to a study done by Kramer et al., who investigated the complications of Le Fort I osteotomy, the overall prevalence rate was about 6% [2]. The risk and extent of these complications increase in patients with anatomical disorders, such as craniofacial dysplasia, orofacial clefts, or vascular anomalies. The risk of ischemic complications is higher in patients requiring extensive dislocations or transverse maxillary segmentation [2]. In this case, the maxillary advancement rate was about 6 mm, and maxillary segmentation was not performed.

Maxillary blood flow was reduced by about 50% within 24 hours after Le Fort I osteotomy if the palatal descending arteries became ligated on both sides [12]. According to Dodson et al.'s study, it returns to normal after one week [13, 14]. In the current study, the arteries on both sides were intact during the operation process, and the collateral perfusion conducted from the soft pedicle was healthy and intact. The following are the four key tenets of palatal fistula correction: large palatal flaps are elevated based on the original incisions, epithelialized fistula margins are removed, the nose and oral mucosa are accurately closed without strain, and the extra tissue is used to repair large or anterior flaws [15].

According to the investigation done by Teemul et al., the maximum soft palatal pedicle stretch was about 10 mm [11], and in this case, both the advancement and manipulation during surgery were less than that.

The patient reported in this study had no local and systemic factors mentioned in Table 1 for maxillary perfusion disorders; therefore, fistula formation cannot be attributed to the above-mentioned factors.

In this patient, the flap was designed as the standard, and no final splint was used. The patient did not mention any post-operative trauma to the area causing necrosis.

Although electrocautery has many benefits, both iatrogenic and patient-related causes can affect its benefits and then cause damage under the following conditions: direct use, insulation failure, direct coupling, and capacitive coupling [16]. Studies have previously shown that having contact with the alveolar bone by active electrocautery leads to time-dependent bone destruction and thermal necrosis of the ablation bone, which does not fill with new bone [17, 18]. In our study, the direct heat and unwanted deep depth of cauterization and its possible contact with the bone to control minor bleeding may have led to bone necrosis of the area as well as the formation of oro-nasal fistula in the incisive papilla. Clear nomenclature is a requirement for fruitful debate, continuous research, and the development of novel therapeutic approaches. The incisive foramen area, the posterior nasal spine, and the uvula are the three most typical sites for fistulas. All post-operative fistulas are discovered to have been caused by the original cleft palate repair either breaking down or failing to heal [19]. Palatal fistulas are a significant concern in the management of cleft palates. Reconstructive surgeons face a difficult task when treating patients with adult cleft lip and palate who have recurrent fistula and skeletal irregularities. Alveolar grafting can stop the remaining fistula and stabilize the arch. The best time to do this is right before the eruption of permanent canines, but tertiary grafting can be done in cases where secondary grafting was not successful or where alveolar clefts persisted into adulthood [19]. After that, prosthodontic reconstruction of the residual cleft site can be done, ideally with an endosseous implant and crown or fixed bridge. Correctly performed, the cleft-orthognathic surgical method enables simultaneous closure of the alveolar fistula, reduction of the cleft-dental gap, and correction of any remaining skeletal irregularities [20]. In a study, 20 of the 636 patients who experienced fistulas have been demonstrated; as a result, the incidence of palatal fistula was 3.1%. The confluence of the hard and soft palates (6/20, 30%), the soft palate (5/20, 25%), and the hard palate itself (9/20, 45%) were the most frequent locations for fistulas. Following cleft palate repair, the palatal fistula incidence was strongly predicted by the cleft palate repair technique [21].

In different people and craniofacial dysplasia, areas of anatomical variations may be seen that the blood supply to which is sensitive to the disruption [22]. The nasopalatine artery, which is disrupted during the Le Fort I osteotomy, may have no established vascular anastomoses in the anterior region with the greater palatine artery, and intraoperative cauterization may be compromised leading to regional necrosis and fistula in this area. For muscular preservation, Santagata et al. have approved that, when a substantial amount of maxillary advancement is needed or when the nasal tip deformity is a concern, the W-shaped osteotomy is a safe surgical procedure. The nasal tip and paranasal region are in perfect preoperative condition. Additionally, because the maxillary ventral movement follows the same lateral maxillary osteotomy lines as in the conventional Le Fort I osteotomy, it is possible to perform as large of maxillary advancements using this technique [23]. Moreover, Rauso et al. have used a pedicled palatal flap technique for surgical repair of small oronasal fistula [24].

## 5. Conclusion

In the patient studied in this research, the mechanism of fistula formation in the incisive papilla was not clearly explained, and the patient had no local and systemic factors causing necrosis.

It is possible that the heat of the electrocautery and its depth as well as the duration of long and unwanted contact with regional tissues, especially bone, can lead to bone necrosis and fistula formation.

On the other hand, with lower probability, the given complete separation of the nasal mucosa from the maxilla, and the vascular and bony anatomical variations in discrepancies, a proper anastomosis with the greater palatine arteries may not be established in the incisive papilla and this may consequently cause avascular necrosis.

## Figures and Tables

**Table 1:** Local and systemic risk factors effective on increasing the risk of improper blood supply of maxilla after Le Fort I osteotomy.

Local	Systemic
Radiation treatment	Cigarette smoking
Infection	Pregnancy
Trauma	Chemotherapy
Surgery related	Haematological conditions
Sacrifice of descending palatine artery	Sickle cell disease
Perforation/stripping palatal mucosa	Leukaemia
Adrenaline injected into mucosa	Gaucher's disease
Perioperative vascular thrombosis	Thalassaemia
Segmental osteotomies	Caisson's disease
Extensive advancement	Systemic lupus erythematosus
Anatomy related	Diabetes mellitus
Craniofacial dysplasia	Vasculitis
Orofacial clefts	Inflammatory bowel disease
Vascular anomalies	Drugs
Previous surgery	Vasoconstrictors
Cleft palate repair	High dose steroids
Surgically assisted rapid palatal expansion	

*Source*. Reprinted from [11].

## Data Availability

The data used to support the findings of this study are included within the article.

## References

[1] Pereira F. L., Yaedú R. Y. F., Sant’Ana A. P., Sant’Ana E. (2010). Maxillary aseptic necrosis after Le Fort I osteotomy: a case report and literature review. *Journal of Oral and Maxillofacial Surgery*.

[2] Kramer F.-J., Baethge C., Swennen G. (2004). Intra- and perioperative complications of the LeFort I osteotomy: a prospective evaluation of 1000 patients. *Journal of Craniofacial Surgery*.

[3] de Mol van Otterloo J. J., Tuinzing D. B., Greebe R. B., van der Kwast W. A. (1991). Intra- and early postoperative complications of the Le Fort I osteotomy: a retrospective study on 410 cases. *Journal of Cranio-Maxillo-Facial Surgery: Official Publication of the European Association for Cranio-Maxillo-Facial Surgery*.

[4] Bendor-Samuel R., Chen Y.-R., Chen P., Chen Y. R. (1995). Unusual complications of the Le Fort I osteotomy. *Plastic and Reconstructive Surgery*.

[5] Wilson M. W., Maheshwari P., Stokes K. (2000). Secondary fractures of Le Fort I osteotomy. *Ophthalmic Plastic and Reconstructive Surgery*.

[6] Siebert J. W., Angrigiani C., McCarthy J. G., Longaker M. T. (1997). Blood supply of the Le Fort I maxillary segment: an anatomic study. *Plastic and Reconstructive Surgery*.

[7] Bell W. H. J. A. J. O. P. A. (1973). Biologic basis for maxillary osteotomies. *Biologic Basis for Maxillary Osteotomies*.

[8] Bell W. H., Fonseca R. J., Kenneky J. W., Levy B. M. (1975). Bone healing and revascularization after total maxillary osteotomy. *Journal of Oral Surgery*.

[9] Ghaly G. A. (2020). Bone healing and revascularisation after total maxillary osteotomy. *50 Landmark Papers Every Oral and Maxillofacial Surgeon Should Know*.

[10] Bell W. (1969). Revascularization and bone healing after anterior maxillary osteotomy: a study using adult rhesus monkeys. *Journal of Oral Surgery*.

[11] Teemul T. A., Perfettini J., Morris D. O., Russell J. L. (2017). Post-operative avascular necrosis of the maxilla: a rare complication following orthognathic surgery. *Journal of Surgical Case Reports*.

[12] Bell W. H., You Z. H., Finn R. A., Fields R. T. (1995). Wound healing after multisegmental Le Fort I osteotomy and transection of the descending palatine vessels. *Journal of Oral and Maxillofacial Surgery*.

[13] Dodson T. B., Bays R. A., Neuenschwander M. C. (1997). Maxillary perfusion during Le Fort I osteotomy after ligation of the descending palatine artery. *Journal of Oral and Maxillofacial Surgery*.

[14] Dodson T., Bays R., Neuenschwander M. (1994). Descending palatine artery ligation and maxillary gingival blood-flow. *Journal of Dental Research*.

[15] Wang Y., Yang F., Liu W., Fan X., Lu Y. (2023). Evaluation of the effect of a resorbable membrane in closure of palatal fistula. *Frontiers in Surgery*.

[16] Wu M.-P., Ou C. S., Chen S. L., Yen E. Y. T., Rowbotham R. (2000). Complications and recommended practices for electrosurgery in laparoscopy. *American Journal of Surgery*.

[17] Azzi R., Kenney E. B., Tsao T. F., Carranza F. A. (1983). The effect of electrosurgery on alveolar bone. *Journal of Periodontology*.

[18] Yoshino T., Aoki A., Oda S. (2009). Long-term histologic analysis of bone tissue alteration and healing following Er: YAG laser irradiation compared to electrosurgery. *Journal of Periodontology*.

[19] Chouairi F., Mets E. J., Gabrick K. S., Alperovich M. (2019). Veau III and Veau IV cleft palate: do peri-operative complications differ?. *Journal of Craniofacial Surgery*.

[20] Tanaka S. A., Mahabir R. C., Jupiter D. C., Menezes J. M. (2012). Updating the epidemiology of cleft lip with or without cleft palate. *Plastic and Reconstructive Surgery*.

[21] Park M. S., Seo H. J., Bae Y. C. (2022). Incidence of fistula after primary cleft palate repair: a 25-year assessment of one surgeon’s experience. *Archives of Plastic Surgery*.

[22] Lanigan D. T., Hey J. H., West R. A. (1990). Aseptic necrosis following maxillary osteotomies: report of 36 cases. *Journal of Oral and Maxillofacial Surgery*.

[23] Santagata M., Sgaramella N., Chirico F. (2022). W-shaped osteotomy to avoid paranasal deformity after standard Le Fort I in orthognathic surgery. *Plastic Surgery*.

[24] Rauso R., Tartaro G., Califano L., Rugge L., Chirico F., Colella G. (2018). Pedicled palatal flap for surgical repair of oro-nasal fistula. *Journal of Biological Regulators and Homeostatic Agents*.

